# The Clustered Regularly Interspaced Short Palindromic Repeat System and Argonaute: An Emerging Bacterial Immunity System for Defense Against Natural Transformation?

**DOI:** 10.3389/fmicb.2020.593301

**Published:** 2020-10-22

**Authors:** Mafeng Liu, Mi Huang, Mingshu Wang, Dekang Zhu, Renyong Jia, Shun Chen, Ling Zhang, Leichang Pan, Anchun Cheng

**Affiliations:** ^1^Institute of Preventive Veterinary Medicine, Sichuan Agricultural University, Chengdu, China; ^2^Research Centre of Avian Disease, College of Veterinary Medicine of Sichuan Agricultural University, Chengdu, China; ^3^Key Laboratory of Animal Disease and Human Health of Sichuan Province, Sichuan Agricultural University, Chengdu, China

**Keywords:** Clustered regularly interspaced short palindromic repeat-Cas, Argonaute proteins, bacterial immunity system, natural transformation, ssDNA

## Abstract

Clustered regularly interspaced short palindromic repeat (CRISPR) systems and prokaryotic Argonaute proteins (Agos) have been shown to defend bacterial and archaeal cells against invading nucleic acids. Indeed, they are important elements for inhibiting horizontal gene transfer between bacterial and archaeal cells. The CRISPR system employs an RNA-guide complex to target invading DNA or RNA, while Agos target DNA using single stranded DNA or RNA as guides. Thus, the CRISPR and Agos systems defend against exogenous nucleic acids by different mechanisms. It is not fully understood how antagonization of these systems occurs during natural transformation, wherein exogenous DNA enters a host cell as single stranded DNA and is then integrated into the host genome. In this review, we discuss the functions and mechanisms of the CRISPR system and Agos in cellular defense against natural transformation.

## Introduction

Horizontal exchange of DNA between bacteria is an important mechanism to generate genome diversity and drive evolution (Gogarten and Townsend, 2005). For example, the emergence of super resistant, virulent bacterial strains has largely been inferred to be caused by the transfer of antimicrobial resistance and virulence genes among different species (Maeusli et al., 2020). The acquisition of genetic material can occur through natural transformation, direct DNA uptake from the environment, conjugation, plasmid transfer from other cells, and transduction, the latter of which incorporates heterologous DNA from bacteriophage infection (Arber, 2014; Darmon and Leach, 2014). Among these, only natural transformation is exclusively facilitated by genes from the bacterial chromosome (Ambur et al., 2016).

Traditionally, the active acquisition of genetic material has been thought to benefit the recipient bacterium. For example, *Streptococcus pneumoniae* required exogenous DNA to become antibiotic resistant and inhibit vaccination treatments (Croucher et al., 2011). However, it was recently suggested that this conclusion was biased because only bacterial genomes that survived selection were ultimately observed as recipients (Ambur et al., 2016). Further, bacteria have been observed to uptake fragmented and damaged DNA by natural transformation when the DNA contains abasic sites or miscoding lesions (Thomas and Nielsen, 2005; Overballe-Petersen et al., 2013). Thus, bacteria indiscriminately take up both “beneficial” and “harmful” DNA. Moreover, these newly integrated gene elements can also alter the structure of the recipient genome and introduce additional physiological burdens (Johnston et al., 2014; Blokesch, 2017).

To control the entry of new genetic material, bacteria have developed immune defenses to limit inter-specific or inter-strain horizontal transfer of chromosomal DNA. Bacterial immune systems, including restriction-modification (R-M) systems, CRISPR systems, and Argonaute proteins (Agos), have recently been observed to play key roles in defending bacterial cells from intrusion of foreign DNA including *via* bacteriophages and plasmid DNA (Bikard et al., 2012; Zhang and Blaser, 2012; Swarts et al., 2014a). However, natural transformation internalizes exogenous ssDNA and integrates it into the host chromosome by homologous recombination. This implies that these immune system mechanisms antagonize natural transformation by unique mechanisms or functions at different stages of natural transformation. For example, R-M systems have been proposed to target natural transformation after the replication of integrated ssDNA into a host chromosome (Johnston et al., 2013).

CRISPR systems were recently shown to inhibit natural transformation in *S. pneumoniae* and *Neisseria meningitidis* (Bikard et al., 2012; Zhang et al., 2013), while the Agos of *Thermus thermophilus* have been shown to prevent the uptake and propagation of naturally transformed plasmid DNA (Swarts et al., 2014a). However, the mechanisms by which CRISPRs and Agos prevent natural transformation has not been fully described in these studies.

## An Overview of the Natural Transformation Process

**Figure 1:**
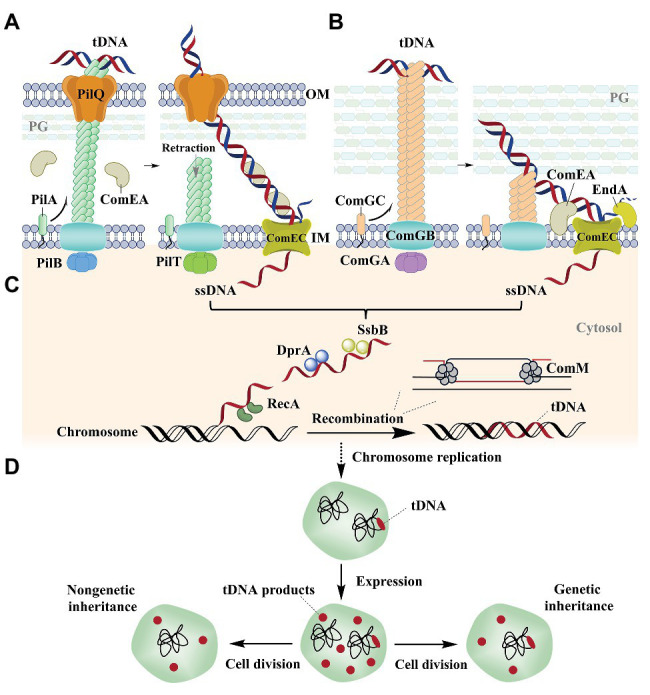
Natural transformation in the Gram-negative bacterium *Vibrio cholerae* (Seitz and Blokesch, 2013; Seitz et al., 2014; Ellison et al., 2018) and the Gram-positive bacterium *Streptococcus pneumoniae* (Laurenceau et al., 2013, 2015; Balaban et al., 2014; Muschiol et al., 2015). **(A)** Natural transformation of *V. cholerae*. Extracellular DNA is bound to the pilus and transported across the OM with the participation of the periplasmic protein ComEA. The pilus is composed of PilA (the main fiber subunit), the PilB and PilT ATPases, PilQ (a secretin pore), PliC (the motor), and other proteins (Matthey and Blokesch, 2016; McCallum et al., 2019). **(B)** Natural transformation of *S. pneumoniae*. DNA is bound to a pseudopilus and is transported across the cell wall with the participation of the membrane protein ComEA. The pseudopilus is mostly composed of ComGC (the major pilin), ComGB (a membrane protein), and ComGA (an ATPase; Muschiol et al., 2015). One strand is degraded by the EndA nuclease, while the other strand is transported into the cytoplasm by ComEC, which also occurs in *V. cholerae.* **(C)** ssDNA integration into the genome. DprA binds internalized ssDNA and recruits the recombinase RecA that integrates ssDNA into the host genome with the help of ComM. **(D)** The replication and expression of the integrated DNA in the cell. After replication of the genome, the host cell contains two genome types. The integrated DNA is immediately expressed in the host cell and the expression product is propagated by both non-genetic and genetic inheritance mechanisms after cell division. tDNA, transforming DNA; M, membrane; OM, outer membrane; IM, inner membrane; PG, peptidoglycan.

Bacterial natural transformation is a complex process involving uptake of extracellular DNA to the cytoplasm and integration into the chromosome. Based on *Helicobacter pylori* (Stingl et al., 2010), *Neisseria* (Maier et al., 2002; Gangel et al., 2014), and *Vibrio cholerae* (Seitz and Blokesch, 2013; Seitz et al., 2014; Ellison et al., 2018) models, Gram-negative bacterial DNA uptake requires its transport across the outer membrane and the translocation of DNA across the inner membrane (Figure 1A).

In natural transformation by *V. cholerae*, the type IV family of pili (T4P) on the surface of most Gram-negative bacteria retracts DNA into the periplasm through direct binding of the extracellular double-stranded DNA (dsDNA) on their ends (Chen and Dubnau, 2004; Claverys et al., 2009; Burton and Dubnau, 2010; Ellison et al., 2018; Figure 1A). Nevertheless, the method by which pili specifically bind DNA remains unclear. It was recently shown that ComE(A), a periplasmic DNA-binding protein, is essential for the uptake and transport of DNA from the outer membrane to the periplasm (Gangel et al., 2014; Hepp and Maier, 2016; Figure 1A). Further, T4P and ComE(A) mediate uptake *via* a Brownian ratchet mechanism (Hepp and Maier, 2016; Dubnau and Blokesch, 2019). An exception to this mechanism has been observed in *H. pylori*, which employs a ComB type-IV secretion system (T4SS), rather than the T4P system, for initial DNA uptake during transformation (Karnholz et al., 2006). ComH is a periplasmic DNA-binding protein that is involved in the transport of DNA into the periplasm, although the interaction between ComB and ComH remains unknown (Damke et al., 2019).

Gram-positive bacteria like *S. pneumonia* use pseudopili to transport extracellular DNA through a thick layer of peptidoglycan (Laurenceau et al., 2013, 2015; Balaban et al., 2014; Muschiol et al., 2015; Figure 1B). In contrast, the Gram-positive bacterium *Bacillus subtilis* initiates DNA binding independent of a pseudopilus (Hahn et al., 2005; Kidane and Graumann, 2005; Mirouze et al., 2018). Rather, it was recently shown that wall teichoic acids (WTAs) are responsible for the initial step in transformation (Mirouze et al., 2018). After the DNA is in the periplasm of Gram-negative cells or in the compartments between the cell wall and the membrane of Gram-positive bacterial cells, one strand is degraded to nucleotides at the membrane surface and the other is internalized into the cytoplasm in single-stranded form through the ComEC transmembrane channel (Draskovic and Dubnau, 2005; Mell and Redfield, 2014; Figures 1A,B). However, the protein that degrades the non-transforming DNA and the mechanism that is used for degradation remains unknown. The internalized single-stranded DNA (ssDNA) is then bound by DNA processing protein A (DprA) that recruits the recombinase RecA to the ssDNA (Mortier-Barriere et al., 2007) and the translocated strand can then be used to replace a chromosomal strand *via* recombination (Figure 1C). During recombination, RecA is responsible for identifying homologous DNA regions and initiates strand invasion to form a displacement loop (D-loop) in the chromosome (Figure 1C). ComM subsequently promotes expansion of the D-loop using a bidirectional helicase and branch migration activities that enhance the integration efficiency of ssDNA into the genome (Nero et al., 2018; Figure 1C). The biological functions and mechanisms of action for ComE(A), ComEC, DprA, RecA, and ComM are all evolutionarily conserved in most competent bacteria (Provvedi and Dubnau, 1999; Berge et al., 2002; Draskovic and Dubnau, 2005; Berge et al., 2013; Johnston et al., 2013; Johnston et al., 2014; Seitz et al., 2014; Hepp and Maier, 2016; Salzer et al., 2016; Nero et al., 2018; Pimentel and Zhang, 2018; Huang et al., 2019).

Following these activities, the integrated DNA is immediately expressed after chromosomal replication, which has been demonstrated to occur before cell division in *V. cholera* (Dalia and Dalia, 2019), *H. pylori* (Corbinais et al., 2016), and *B. subtilis* (Boonstra et al., 2018; Figure 1D). Thus, the expressed products of integrated DNA can be used by the untransformed relatives of transformed cells, which are termed the non-genetic inheritance of traits (Dalia and Dalia, 2019; Figure 1D).

## The Crispr System and Natural Transformation

### An Overview of CRISPR Systems

Jansen et al. (2002) first discovered clustered regularly interspaced short palindromic repeats (CRISPR) loci and the CRISPR-associated Cas genes. The genomes of ∼50% of bacteria and ∼90% of archaea have a CRISPR-Cas system that plays a role in defense against the inclusion of foreign (e.g., phage or plasmid) DNA (Makarova and Koonin, 2015; Makarova et al., 2015; Samson et al., 2015). CRISPR loci are composed of ~24–48 bp short repetitive sequence arrays that are separated by equally short “spacer” sequences that are derived from mobile genetic elements like bacteriophages and plasmid sequences (Mojica et al., 2005). CRISPR-Cas systems have been divided into two distinct classes: class 1 and class 2. Class 1 systems have multi-subunit effector complexes, whereas class 2 systems have individual single-protein effector modules (Koonin et al., 2017; Koonin and Makarova, 2019). Furthermore, class 1 systems contain three different sub-types (types I, III, and IV), while class 2 systems contain types II, V, and VI. Each sub-type is characterized by distinct effector module architectures that contain unique signature proteins, like Cas3 for the type I systems, Cas9 for type II systems, and Cas10 for type III systems (Makarova and Koonin, 2015; Koonin et al., 2017; Koonin and Makarova, 2019).

CRISPR-Cas systems operate through three steps. In the first (the adaptation phase), the acquisition of spacers is often derived from phage or foreign plasmid DNA (Mojica et al., 2005). Importantly, the 2–5 nt protospacer adjacent motif (PAM) in the invading DNA is required for spacer acquisition (Marraffini and Sontheimer, 2010a). During adaptation, the Cas1-Cas2 complex is also required to process foreign DNA and subsequent integration into a CRISPR array (Hille et al., 2018). In the second step, short CRISPR RNAs (crRNAs) undergo biogenesis and maturation. In most bacteria, the repeat/spacer arrays are transcribed as long CRISPR RNA precursors (pre-crRNA) that are then cleaved within the repeat sequences and transformed into small crRNAs by Cas endoribonucleases (Hatoum-Aslan et al., 2011), which then base pair with foreign DNA *via* spacer-encoded sequences (Brouns et al., 2008). In class 1 systems, Cas-6-family enzymes are involved in processing RNA into mature crRNAs, while Cas9 is involved in the maturation of crRNAs in class 2, type II systems (Hille et al., 2018). The third step is the interference phase, wherein crRNAs are used as antisense guides that combine with sets of Cas proteins to form the core CRISPR-Cas ribonucleoprotein complexes. After complementary “protospacer” sequences from foreign invading DNA or RNA are recognized by these complexes, they are cleaved *via* sequence-specific mechanisms (Garneau et al., 2010). The PAM of invading DNA is also required for efficient CRISPR interference (Fischer et al., 2012). Further, crRNA-Cas complexes are base-paired with PAM sequences to avoid autoimmunity (Marraffini and Sontheimer, 2010b). Thus, type I and type II CRISPR-Cas systems cleave DNA, while type III systems can cleave DNA or RNA molecules (Barrangou and Marraffini, 2014). Specifically, *cas9* of type II CRISPR-Cas systems encodes a multidomain protein that contains all the functions of effector complexes and targets DNA cleavage sites (Jinek et al., 2012). Based on the above observations, the CRISPR system has been described as an RNA-guided “adaptive immune system” of bacteria. In addition, CRISPR-Cas systems also exhibit off-target activities on sequences that are similar to target sequences (Nivala et al., 2018), which may represent a potential “escape” mechanism for foreign DNA.

### The CRISPR System Antagonizes Natural Transformation

Numerous studies have demonstrated that CRISPR systems function in defense against bacteriophage infection and plasmid transformation (Garneau et al., 2010; Amitai and Sorek, 2016). Accordingly, most archaeal spacers correspond to plasmids or bacteriophages (Brodt et al., 2011). Further, some of these sequences can be mapped to the chromosomal genes of other archaea, suggesting that CRISPR/Cas systems may also be involved in reducing the intrusion of foreign chromosomal DNA *via* natural transformation (Brodt et al., 2011). Bikard et al. (2012) introduced the CRISPR01 locus of *Streptococcus pyogenes* SF370 into the non-encapsulated strain, *S. pneumoniae* R6. The chromosomal DNA of the *S. pneumoniae* strain carrying the engineered spacer 1 (*spc1*) target was used as the donor for transformation with *S. pneumoniae* R6 and the *S. pneumoniae* crR6 strains. The *S. pneumoniae* crR6 strain prevented DNA transformation, while the control *S. pneumoniae* R6 strain could not prevent transformation (Bikard et al., 2012). Furthermore, a spacer for the *cap* gene was introduced into the CRISPR locus of *S. pneumoniae* crR6, and CRISPR interference was able to prevent capsule-switching of pneumococci both *in vitro* and *in vivo* (Bikard et al., 2012). Thus, CRISPR/Cas systems can prevent natural transformation, at least in *S. pneumoniae*. Nevertheless, it is unclear if native CRISPR/Cas systems can limit natural transformation, since all known published pneumococcal genomes do not encode CRISPR loci (Makarova et al., 2011).

Zhang et al. (2013) subsequently cloned protospacers with flanking *Neisseria* sequences that conform to the PAM consensus sequence into the pGCC2 plasmid. It was then shown that the plasmid pGCC2 can be integrated into the genome of *N. meningitidis* through natural transformation. However, a plasmid containing protospacers failed to integrate into *N. meningitidis* through natural transformation, in contrast to the empty plasmid (Zhang et al., 2013). Mutation of two consecutive nucleotides within the PAM or seed sequence of the protospacer in the plasmid abolished CRISPR interference, thereby reestablishing the natural transformation potential. In contrast, nucleotide substitutions in a non-PAM flanking region or substitutions in non-seed protospacer positions did not affect CRISPR interference (Zhang et al., 2013). Similarly, natural transformation was inhibited when using genomic DNA containing protospacers. The CRISPR-Cas systems in both of the above studies were type II systems that use Cas9 proteins as effector proteins to cleave invading nucleic acids (Makarova et al., 2015).

### Evolutionary Analysis of Natural Transformation and CRISPR Systems

The evolutionary association between natural transformation and CRISPR systems has been investigated through comparative genomics. The loss of competence in *Aggregatibacter actinomycetemcomitans* was strongly and positively correlated with the loss of a CRISPR system (Jorth and Whiteley, 2012). Moreover, the genomes of competent bacteria were larger and contained multiple rearrangements in contrast to the non-competent strain genomes. Rather, non-competent bacterial genomes were extremely stable, but susceptible to infective DNA element integration (Jorth and Whiteley, 2012), suggesting that CRISPRs play a role in defense against exogenous DNA invasion.

Using the length of a CRISPR array as a proxy for CRISPR activity, Gophna et al. (2015) analyzed the connection between CRISPR activity and gene acquisition *via* horizontal gene transfer (HGT). CRISPR-negative bacterial genomes encoded fewer prophage-encoded proteins on average compared to CRISPR-positive genomes, suggesting that CRISPR systems do not inhibit HGT on evolutionary timescales (Gophna et al., 2015). It was instead hypothesized that the resistance of CRISPR-Cas systems to mobile elements occurs at the population scale rather than over evolutionary timescales (Gophna et al., 2015).

The Csx27 protein of subtype VI-B1 CRISPR-Cas systems was recently shown to be encoded in the same predicted operons as the components of natural transformation systems (Makarova et al., 2019). The Csx27 protein has four predicted transmembrane regions, and it was thus predicted that Csx27 proteins form membrane channels for the transport or degradation of ssDNA (Makarova et al., 2019). Taken together, the interactive relationships between natural transformation and CRISPR systems cannot be adequately established through evolutionary analysis. Nevertheless, the connection between CRISPRs and natural transformation requires further experimental investigation.

### The Unstated Conundrum and a Proposed Model for CRISPR System Limiting of Natural Transformation

**Figure 2:**
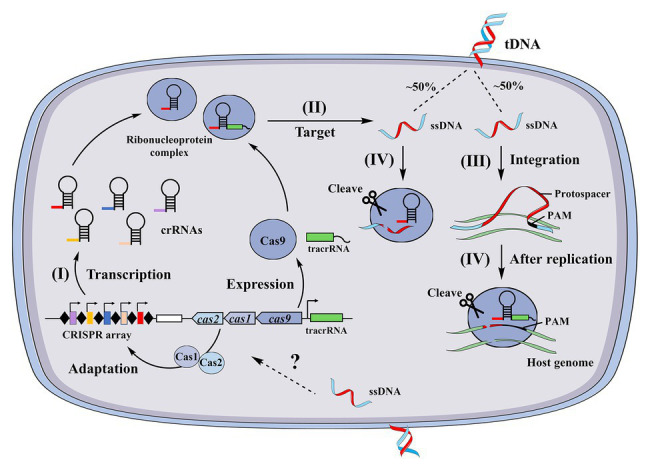
A proposed model of type II clustered regularly interspaced short palindromic repeat (CRISPR) system inhibition of natural transformation. CRISPRs consist of short repeats separated by non-repetitive spacers that are obtained from previous invading sequences. (I) CRISPR loci are transcribed as long CRISPR RNA (crRNA) precursors and then cleaved into small mature crRNAs, (II) during natural transformation, heterologous DNA enters the cell as ssDNA that is then targeted by the CRISPR system, (III) after replication in the transformant chromosome, a dsDNA that contains a “protospacer” and PAM sequence is produced, and (IV) the complex of crRNAs and Cas proteins recognize and cleave the complementary “protospacer” in the transformed chromosomes.

Although the above two studies suggested that CRISPR systems can limit natural transformation, several associated conundrums have not been resolved. First, it is unclear whether and how new spacers are acquired from internalized ssDNA. Second, it is unknown if CRISPR systems target internalized ssDNA or dsDNA after exogenous genes are integrated and replicated into the host genome. To our knowledge, no reports indicate that CRISPR systems can acquire spacers from ssDNA. The RNA guided Cas9 proteins from *N. meningitidis* and *S. pyogenes* were recently shown to cleave ssDNA in a PAM- and tracrRNA-independent manner (Ma et al., 2015; Zhang et al., 2015), suggesting that the CRISPR systems of *N. meningitidis* and *S. pyogenes* target ssDNA. In addition, Cas12a of Lachnospiraceae and the Cas1 protein of *Escherichia coli* (Babu et al., 2011) have been observed to target ssDNA and degrade it (Chen et al., 2018). A model was proposed for the antagonization of heterologous transformation by CRISPR systems in combination with the proposed post-replication targeting model for CRISPR systems (Johnston et al., 2013) using the *Neisseria* CRISPR system as an example (Figure 2). In the model, (I) spacers are pre-acquired from internalized ssDNA through an unidentified pattern and (II) heterologous dsDNA enters the cytoplasm as ssDNA through natural transformation. About 50% of ssDNA is targeted and cleaved by the crRNA and Cas9 endonuclease complexes in this stage, since each strand of the dsDNA randomly enters the cell. Thus, a portion of ssDNA escapes this immune response, and (III) after integration and replication, the double-stranded DNA associated with a PAM is produced in the transformant chromosome. At this stage, the CRISPR system in the host genome can target and cleave the genomic PAM site, but the CRISPR system cannot target itself, as these regions lack a PAM site (Johnston et al., 2013; Figure 2). In support of this hypothetical model, the transfer of active CRISPR/Cas systems into a recipient cell containing a target sequence has been shown to result in cell death (Bikard et al., 2012). This model is based on the requirement of a spacer to pre-exist in the host genome and coincide with the protospacers of invading DNA. Nevertheless, the fundamental question as to whether native CRISPR/Cas systems acquire new spacers from internalized ssDNA remains unaddressed. Another important question that remains to be answered is whether other types of CRISPR/Cas systems are involved in antagonizing natural transformation.

## Argonautes Inhibit Natural Transformation

Argonaute proteins (Agos) were initially discovered in eukaryotes and were later observed as conserved across all domains of life (Bohmert et al., 1998; Hock and Meister, 2008). Agos bind small noncoding RNAs in eukaryotes and target complementary RNA to regulate gene expression and repress invasive genomic elements (Peters and Meister, 2007). The homologues of Argonautes are present in some bacterial (~9%) and archaeal (~32%) genomes (Makarova et al., 2009; Swarts et al., 2014b). However, bacterial and archaeal Agos do not encode Dicer homologs and the TAR RNA-binding protein (TRBP) that is important for the silencing pathway.

### Argonaute Interference With the Replication of Exogenous Plasmids Internalized by Natural Transformation

**Figure 3:**
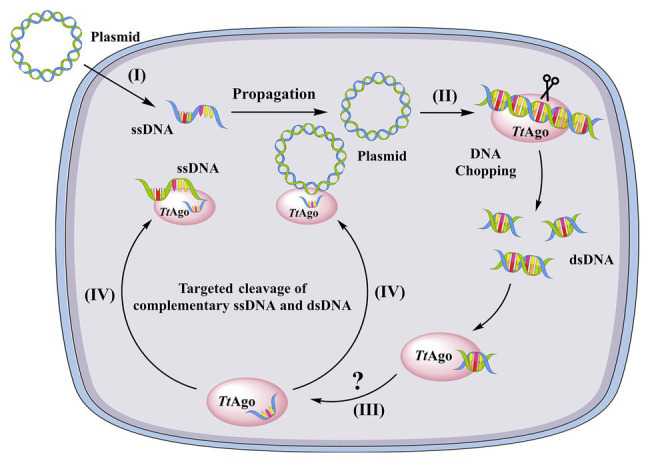
A model of TtAgo-mediated DNA-guided plasmid inhibition. (I) a plasmid enters the cell as ssDNA through natural transformation. At this stage, the ssDNA is resistant to apo-TtAgo, (II) after replication, the dsDNA plasmid is chopped by apo-TtAgo, (III) the fragmented dsDNA is then bound by TtAgo and one strand of the DNA is released from TtAgo through an unknown mechanism, and (IV) the siDNA-loaded TtAgo targets complementary DNA (ssDNA and dsDNA) and cleaves it.

The domain organization of some prokaryotic Argonautes was observed to be similar to eukaryotic orthologs (Song et al., 2004; Yuan et al., 2005; Wang et al., 2008). However, archaeal and bacterial Argonautes have a higher affinity for ssDNA and dsDNA compared to eukaryotic homologs (Ma et al., 2005; Yuan et al., 2005). Nevertheless, experimental evidence to understand the functions of prokaryotic Argonautes in host defenses have not appeared until recently. *ago* mutation in *T. thermophilus* leads to increased natural transformation efficiency of plasmids by 10-fold compared to wild-type cells (Swarts et al., 2014a). Moreover, plasmid yields from wild-type cells are lower than those of *ago* knockout strain (Swarts et al., 2014a). Analysis of co-purified nucleic acids revealed that the Ago from *T. thermophilus* (TtAgo) binds 13–25 bp ssDNAs that are mostly derived from plasmids and have a strong bias for the 5′-end deoxycytidine (Swarts et al., 2014a). Plasmid cleavage assays also showed that guide DNA-loaded TtAgo was able to cleave both single- and double-stranded targets (Swarts et al., 2014a). Thus, the authors speculated that TtAgo uses siDNA guides to specifically cleave ssDNA targets that are produced during natural transformation (Swarts et al., 2014a; Figure 3). Recent studies have suggested that most characterized Argonautes from bacteria and archaea function to target complementary dsDNA or ssDNA against invasive genetic elements, in contrast to their functions in eukaryotes (Makarova et al., 2009; Olovnikov et al., 2013; Swarts et al., 2014a). Agos bind small RNAs (15–19 nt) in *Rhodobacter sphaeroides* that are derived from mRNAs or are the products of their degradation (Olovnikov et al., 2013). In addition, Olovnikov et al. (2013) observed strong degradation of a plasmid upon the expression of RsAgo in *E. coli* cells, although Argonaute-dependent cleavage activity was not detected. Thus, it was proposed that RsAgo use RNA guides to recruit an associated nuclease for subsequent target cleavage (Olovnikov et al., 2013).

### The DNA Cleavage Mechanism of Bacterial and Archaeal Argonautes

A remaining question was how guiding DNAs were generated and loaded onto Agos in bacterial cells. Guide-free TtAgo, SeAgo, and MjAgo were shown to degrade double-stranded plasmid and genomic DNA to 8–26 nt oligonucleotides, an activity that was termed “DNA chopping,” with the subsequent small dsDNA fragments loaded onto Agos (Swarts et al., 2017; Zander et al., 2017; Olina et al., 2020). Moreover, the cleavage efficiency of pre-loaded MjAgo for a plasmid was higher when compared to apo-MjAgo (Zander et al., 2017). Similarly, the use of cleavage products as functional guides was also demonstrated for TtAgo (Swarts et al., 2017). In addition to TtAgo, several bacterial and archaeal Argonautes have been shown *in vitro* to cleave target DNA using ssDNA as a guide, including the Argonaute from *Pyrococcus furiosus* (PfAgo; Swarts et al., 2015), the Argonaute of *Clostridium butyricum* (CbAgo; Hegge et al., 2019; Kuzmenko et al., 2019), the Argonaute of *Limnothrix rosea* (LrAgo; Kuzmenko et al., 2019), the Argonaute of *Methanocaldococcus jannaschii* (MjAgo; Zander et al., 2017) and the Argonaute of *Synechococcus elongatus* (SeAgo; Olina et al., 2020). Additionally, the Argonautes in some bacteria, such as *Marinitoga piezophile*, *Thermotoga profunda*, and *Rhodobacter sphaeroides*, use RNA as a guide to target DNA (Kaya et al., 2016; Miyoshi et al., 2016).

### A Proposed Model and Remaining Conundrums of the Inhibition of Natural Transformation by Argonautes

In summary, a scenario can be envisioned wherein pAgo, using TtAgo as an example, limits natural transformation when a plasmid is the substrate DNA, as shown in Figure 3. In this generalized mechanism, (I) a plasmid enters a cell by natural transformation as ssDNA that is resistant to the “chopping activity” of TtAgo since the guide-free TtAgo cannot degrade ssDNA (Swarts et al., 2017), (II) after propagation, the plasmid DNA is chopped by apo-TtAgo and the small DNA fragments are loaded into TtAgo (Figure 3), and (III) the target strand is then dissociated from TtAgo through unknown mechanisms and TtAgo-siDNA attacks complementary ssDNA and dsDNA (Figure 3). However, unanswered questions remain regarding these mechanisms. First, if TtAgo has both “DNA chopping” and ssDNA guided cleavage activities, then all transformed plasmids should be cleaved. However, un-cleaved plasmids remain within *T. thermophilus* (Swarts et al., 2014a). Second, it is unknown how endogenous plasmids coexist with TtAgo and also how small DNA-loaded Ago complexes distinguish target foreign DNA from normal genomic substrates. Even over-expressed Ago in *S. elongatus* did not affect cell growth (Olina et al., 2020), suggesting that Ago does not disrupt the genome of *S. elongatus*. Further, it has been proposed that *M. jannaschii* histones, but not methylation, protects genomic DNA from Ago chopping activities (Zander et al., 2017). However, the *T. thermophilus* genome does not encode histones (Willkomm et al., 2018). An additional question is how one of the DNA strands released from Ago binds to dsDNAs. Lastly, it is unknown if Ago limits natural transformation when using exogenous DNA fragments as the substrate. It was recently shown that most SeAgo-associated small DNAs were derived from the proposed genomic replication initiation and termination sites (Olina et al., 2020). Therefore, it is possible that Agos target exogenous DNA when forming DNA intermediates during host cell replication.

## Conclusions and Perspectives

The nucleic acid-guided binding and cleavage activities of pAgos are reminiscent of CRISPR-Cas systems. Like CRISPR-Cas systems, pAgos degrade invading DNA into short dsDNA fragments. However, unlike CRISPR-Cas systems, pAgo-mediated defenses do not integrate degraded fragments into host genomes and, thus, no memory of the invading DNA is generated. Therefore, pAgos can be described as the “innate” immune systems of bacteria and archaea (Hegge et al., 2018; Kuzmenko et al., 2019), while the CRISPR-Cas systems can be described as “adaptive” immune systems. The innate and adaptive immune systems interact in mammals, although it is unknown if such interactivity occurs between CRISPR-Cas systems and pAgos. The *ago* genes of *M. piezophila*, *M. kandleri*, and *T. profunda* are present in the same operon as the *cas* gene of the CRISPR-Cas locus (Kaya et al., 2016), although most bacterial and archaeal genomes that encode CRISPR-Cas loci lack Argonaute genes (Makarova et al., 2006). Thus, the potential interrelationships of Ago and CRISPR-Cas systems require further experimental demonstration.

## Author Contributions

ML, MH, MW, and AC conceived and designed the manuscript. ML, MH, RJ, and SC wrote the paper. MH, DZ, and LZ contributed the figures. ML, MH, and LP supervised the studies and corrected the manuscripts. All the authors have reviewed the manuscript. All the authors who contributed to this work have read the manuscript and approved publication.

### Conflict of Interest

The authors declare that the research was conducted in the absence of any commercial or financial relationships that could be construed as a potential conflict of interest.
